# The “Haves, Have Some, and Have Nots:” a Latent Profile Analysis of Capacity, Quality, and Implementation in Community-Based Afterschool Programs

**DOI:** 10.1007/s11121-021-01258-z

**Published:** 2021-06-30

**Authors:** Emilie Phillips Smith, Dawn P. Witherspoon, Pui-Wa Lei

**Affiliations:** 1 Michigan State University, East Lansing, MI 48824 USA; 2 The Pennsylvania State University, State College, USA

**Keywords:** Afterschool programs, Program quality, Capacity, Implementation, Evidence-based practices, Pax Good Behavior Game, Latent profile analysis, Neighborhood/community contexts, racial-ethnic, geographic, socio-economic diversity

## Abstract

Implementation of evidence-based practices is a critical factor in whether afterschool programs are successful in having a positive impact upon risk reduction and positive youth development. However, important prevention research reveals that contextual and organizational factors can affect implementation (Bradshaw & Pas in School Psychology Review, 40, 530–548, 2011) (Flaspohler et al., in American Journal of Community Psychology, 50(3-4), 271-281, 2012) (Gottfredson et al., Prevention Science, 3, 43–56, 2002) (McIntosh et al., Journal of Positive Behavior Interventions, 18(4), 209-218, 2016) (Payne in Prevention Science, 10, 151–167, 2009). Using a latent profile approach (LPA), this paper examines multiple organizational and neighborhood contextual factors that might affect the degree to which afterschool programs effectively implement evidence-based practices in the context of a cluster-randomized trial of the Paxis Good Behavior Game (PaxGBG). The Interactive Systems Framework (ISF) explores dimensions of capacity that might matter for prevention efforts. As expected, we found that well-resourced and high-quality programs performed well in terms of implementation (the Haves) and, in neighborhood contexts rich in racial-ethnic diversity. Yet, we found that some programs with less physical and material capacity (the Have Nots), demonstrated greater program quality (i.e., supportive adult and peer relationships, engagement, a sense of belonging) *and* implementation, relative to programs with better capacity (e.g., space, material resources, staffing, and leadership, the Have Somes). While capacity matters, intentional prevention initiatives that seek to promote evidence-based practices are helpful to sites in supporting organizations that might otherwise fail to provide quality programming for youth. This paper addresses a conundrum in prevention science, namely, how to make programming accessible to those who need it with a focus on organizational processes, program quality, and implementation of evidence-based practices.

## The Salience of Quality Community-Based Afterschool Programs

Increasingly over the past two decades, community-based afterschool programs (ASPs) have been recognized as important contexts in the lives of developing youth. These programs were popularized by the 21st Century (21C) Community Learning Centers, a federal act intended to support working families, extend school day learning, and provide opportunities for engaging interactions for children with supportive adults and peers (Afterschool Alliance, 2014). With the growth of dual-career families and working parents, the afterschool hours from 3 to 7 pm emerged as the riskiest of a student’s day with increased delinquency and substance abuse (Snyder & Sickmund, 2006; Taheri & Welsh, 2016). The intent of 21C was to support community-based ASPs in their important role in keeping youth in safe, supportive, appropriately structured, and engaging environments. These programs are often physically located in schools or community settings, staffed, and managed by schools or a range of youth development organizations such as the YM/YWCA, the Boys and Girls Club, local parks and recreation commissions, or private caregiving organizations.

Over the past few decades, educators, social scientists, and policymakers have increasingly recognized the role of ASPs in improving children’s academic, socio-emotional outcomes, and relational experiences (Cross et al., 2010; Hall et al., 2003; Kuperminc et al., 2019; Mahoney et al., 2009; Riggs & Greenberg, 2004; Roth, et al., 2010). Children who participated in ASPs using evidence-based practices were more likely to report a sense of competence and pride in school, handle anger in socially appropriate ways, exhibit better academic attendance and performance, develop more positive social identities, and demonstrate more positive youth development (Belgrave et al., 2004; Durlak et al., 2010; Scott-Little et al., 2002; Smith et al., 2018; Taylor et al., 2017; Tebes et al., 2007; Vandell et al., 2018).

However, the findings regarding ASP’s are not all so positive; a national study of twenty-first century centers found mixed and null effects of afterschool participation. Though participants felt safer, gains in academic and social development were reported only for those lower in academic achievement and few behavioral benefits were detected (James-Burdumy et al., 2007); later work essentially replicated these findings (Gottfredson et al., 2010). However, the question of whether variations in quality of programming contributed to these findings was unanswered. Other null effects were reported in a meta-analysis categorizing programs by whether the focus was primarily academic, recreational, or skills-training; no program type significantly reduced delinquency though no harmful effects were evident (Taheri & Welsh, 2016). On the other hand, the evidence does seem clear that afterschool settings with little structure or monitoring actually contribute to deviancy training (Mahoney et al., 2004; Rorie et al., 2011). Thus, the findings on the efficacy of afterschool programs in reducing problem behavior and promoting positive adjustment and achievement are mixed.

The difference between programs with successful versus neutral effects is likely due, at least in part, to the quality of the program. Lauer et al. (2006) found several aspects of the afterschool program influenced findings, for example, working with students one-on-one, a focus on reading for elementary students and math for secondary students, and competent supervisors characterized the programs with more benefits for youth. Further, youth who attended highly structured “SAFE” programs (i.e., programs that were sequenced, active, focused, and explicit), utilizing evidence-based practices (EBPs), were significantly more likely to demonstrate growth in multiple outcome domains (Durlak et al., 2010; Taylor et al., 2017). Certain aspects of afterschool programs have been found to affect children’s outcomes such as more engaging learning strategies, supportive capable adults, appropriate structure coupled with strategies that foster positive behavior, and self-regulation (Cross et al, 2010; Lauer et al., 2006; Smith et al., 2018) and even co-regulation is a prospect in collaborative learning settings (Volet et al., 2009).

## The Role of Implementation and Context in Prevention Science 

Given the importance of key ingredients in afterschool practices, the focus turns to fostering implementation. An important body of prevention science has focused on exploring the degree to which implementation and efficacy vary across a spectrum of factors, including the contexts of where the work is done. The following discussion draws upon research with families and schools that may be instructive for afterschool.

### Implementation and Efficacy

 Substantial research has attended to the important role of implementation exposure, also referred to as dosage, in treatment efficacy. An example of the role of implementation dosage is found in the Multi-Site Violence Prevention Project, a cluster randomized trial across 4 universities and 37 school districts (Simon and the MVPP, 2009). The MVPP included both a universal school intervention focused on increasing cognitive behavioral self-regulation and a selective intervention that was delivered to families of youth who were rated as high risk for aggression *and* influential among their peers. Intent-to-treat effects between the intervention and control conditions were not detected. However, when implementation dosage, namely participation in the family programs was accounted for, effects were found upon youth aggression and value for educational achievement that was mediated through change in parent disciplinary practices and family cohesion (Henry and the MVPP, 2011, 2012; Smith et al., 2004). The selective family-based intervention with influential albeit more aggressive students resulted in less school-wide aggression, supportive of an ecological-social networks effect. The critical role of implementation dosage has been demonstrated in research with families and schools (Huang et al., 2014; Kogan et al., 2019; Lochman et al., 2006).

### Neighborhood and Community Contexts Matter

 While implementation is frequently acknowledged as critical, community context is another potentially moderating factor considered in prevention science. An ecological-developmental perspective acknowledges a myriad of family, neighborhood, and community contexts that impact development (Eron et al., 2002; Leventhal & Brooks-Gunn, 2000; Sampson, et al., 2002; Smith et al., 2004; Witherspoon et al., 2016). Conceptually, social disorganization theory suggests that neighborhood structural factors (e.g., residential instability, poverty, ethnic/minority status, and single parent households) might make prevention approaches more challenging, requiring substantial resources to recruit, retain, and engage participants. Alternatively, social capital/collective socialization models go beyond structural factors to focus on social ties and connections within a community that may help promote positive outcomes (Ainsworth, 2010; McKnight & Kretzman, 1996). The role of community context in prevention is an important factor to consider.

Context has been explored in a notable, longitudinal prevention study spanning multiple states and 9000 participants, including a multi-component family, peer, school, and community approach. Effects were found upon both teacher- and parent-reported conduct problems, social skills, and official indices of severity of arrests as late as adulthood (Conduct Problems Prevention Research Group, 2010). Yet, with these impactful, statistically and socially significant findings, geographic sites differed in the persistence of effects upon adult arrests with some urban sites demonstrating reduced impact. Similarly, violence prevention programming has been found to be more effective in reducing aggression using family and school approaches in less disadvantaged school communities while impoverished urban communities were found to exhibit heightened levels of aggression (Eron et al., 2002). In the follow-up research to this study, the experimental group in higher-risk urban settings revealed effects that approached but did not attain statistical significance (Tolan et al., 2020). Reducing aggression has been more difficult in neighborhood contexts where youth must face and contend with chronic poverty, unemployment, discrimination, and violence, reflecting the existing inequality that complicates the lives of the participants and the prevention efforts (Sampson, 2008). Because of the persistence of inequality, prevention science needs to address systemic issues more fully in current and future work.

Based upon these varying conceptual frameworks, it is plausible that extreme risk and disadvantage may overwhelm prevention approaches or one might expect that more change is demonstrated in communities with more need but available social capital. The question is whether existing neighborhoods have or can be infused with assets beneficial to prevention efforts and whether these settings can effectively capitalize upon these resources.

## Capacity and Organizational Factors in Prevention Implementation

Though we have learned much about the characteristics of quality afterschool programs, we know less about how capacity and context might interact with program implementation in these settings though some funders, like the Wallace Foundation, have launched national initiatives to strengthen support for afterschool programming (Yohalem et al., 2012). There is a growing literature which indicates that *capacity*, factors such as organizational climate, communication, clearly articulated goals and activities, multi-level decision-making processes, and leadership styles play an important role in the implementation and sustainability of evidence-based practices (Birken et al., 2017; Schoenwald & Hoagwood, 2001; Spoth et al., 2015; Williams et al., 2017). Given the key role of implementation science that explores adoption of and sustaining best practices, explicating the contributions of capacity could prove enlightening (Bertram et al., 2015; Elias et al., 2003; Elliott & Mihalic, 2004).

The Interactive Systems framework (ISF) proposes multi-level factors that can impact implementation of evidence-based practices (Wandersman et al., 2008). *General capacity* broadly refers to organizational factors such as program structure, communication, management, mission and vision, and activities related to maintaining a functioning unit (Domitrovich et al., 2008; Flaspohler, Lesesne, et al., 2012). There is also a community aspect of general capacity entailing linkages to children’s families, schools, and other organizations that provide tangible and social resources; both organizational and community aspects of general capacity have been found to be related to implementation in afterschool (Halgunseth et al., 2012). *Innovation-specific capacity* examines the ability to integrate new methods or practices into an organization. Theoretical models of change can help delineate the processes by which various factors either help or hinder implementation and treatment efficacy (Hasson, 2010; Lipsey & Cordray, 2000).

Increasingly, researchers are recognizing that organizations may vary in the degree to which they can feasibly support quality implementation with less-resourced settings facing more challenges in this regard (McIntosh, et al., 2016). In contrast, other studies have found that schools serving needier minority populations are *more* likely to select and integrate standardized behavioral programing (Payne, 2009) and that they are more likely to receive training and adopt programming than schools with higher levels of academic achievement (Bradshaw & Pas, 2011). The concept of “readiness” of an organization to implement evidence-based practices has emerged as an important factor (Flaspohler, Meehan, et al., 2012; Tseng & Seidman, 2007). For example, individual educator willingness has been found to impact effective prevention efforts (St. George, 2016). Effective implementation of EBP’s has been found in organizations with supportive leadership, openness to change, and communication among staff and leadership (Chilenski, et al., 2007, 2015).

## Fostering Afterschool Quality and Implementation of Evidence-Based Practices 

As discussed earlier, a range of youth-serving organizations is involved in providing afterschool programming. The research in ASPs has been quite attuned to the degree to which quality, conceptualized as engaged learning experiences with supportive adults and peers in appropriately structured programs, fosters positive developmental outcomes (Eccles and Gootman, 2002; Little et al., 2008). Over the past 12 years, the research team has been collaborating with afterschool programs to implement evidence-based practices. Specifically, the Pax Good Behavior Game (PaxGBG) is an ecological intervention designed to create shared behavioral norms and practices that promote an environment conducive to learning, and socio-emotional regulation (Embry, 2002). PaxGBG was adapted from the original Good Behavior Game (GBG) developed in the 1960s (Barrish et al., 1969), which has been shown to reduce early aggression and substance abuse in youth and early adults (Kellam et al., 2008; Flower et al., 2014). PaxGBG is a cooperative game played for only a few minutes at a time among heterogeneous teams of children engaged in simple tasks, which allow teams to earn activity rewards when they limit disruptive behavior. Embry describes this as a process that “thanks youth for not [misbehaving],” with praise and encouragement for group co-regulation. As students improve, it is played for longer periods, during different activities, times of day, with variable opportunities for earned activity rewards. The game is combined with “kernels,” that is posted reminders and signals, creating a collection of nurturing strategies to foster “co-regulation” with youth participants and adults monitoring and praising each other (Smith & Bradshaw, 2017). In our migration of PaxGBG into afterschool, findings from our cluster-randomized trial indicated that with better implementation, higher quality was observed—staff were observed as less harsh and critical, more supportive and engaging, and children reported less hyperactivity and more prosocial behavior (i.e., sharing, caring, and listening). With implementation and quality, children evidenced more positive youth development including competence, connectedness, and character, and another potential dimension of positive youth development (PYD), the cultural value of respect for adults, was exhibited particularly among the African-American and Latino students (Smith et al., 2017, 2018).

However, what remains to be tested is whether PaxGBG works equally well in all types of programs or if additional supports are needed. Implementation science examines the range of organizational and external factors that potentially impact the adoption and utilization of evidence-based practices (Bertam et al., 2015; Kallitsoglou, 2020; Novins et al., 2013; Williams et al., 2017). Grounded in the ISF discussed earlier (Wandersman et al., 2008), our earlier work explored ways to characterize a small group of programs in terms of capacity and relationships to our measures of implementation among an early cohort of subsampled programs (Flaspohler, Lesesne, et al., 2012; Flaspohler, Meehan, et al., 2012; Halgunseth et al., 2012). In this study involving all our participating programs across 3 successive cohorts/years, we sought to further operationalize dimensions of capacity with attention to program organization, staffing, children served, *and* the neighborhood contexts of these programs, to examine relationships to implementation.

Over the past several years, our work has been conducted with a cadre of youth-serving programs that vary in terms of geographic locale, defining characteristics of the programs and staff, and the racial-ethnic background and socio-economic status of the children served. As such, we sought to use a methodology that would respect the value of diversity and the fact that no one size necessarily fits all; there are multiple pathways to success. With this idea, we choose to use a more person-, or in this context, program-centered versus variable-centered approach. The idea of multifinality suggests that any one factor may function differently depending on the context in which it operates (Cicchetti & Rogosch, 1996; Yu et al., 2019). In doing so, we would be able to explore heterogeneity (Lanza & Cooper, 2016) in the various configurations of the programs.

## Summary and Rationale for the Present Study

In sum, afterschool programs have demonstrated positive effects upon youth behavior and achievement, but quality and capacity are likely key. Leadership, staffing models, and leveraging available community resources may be important processes along with more tangible aspects of capacity such as staffing, physical space, and materials. Further, based upon an eco-developmental model and the ISF, we know less about how organizational and neighborhood contexts affect afterschool programs. Afterschool programs are nested in neighborhood contexts that may impact their functioning. As such, our study examines the following research questions:

1. Can we describe different types of programs based on their general capacity (i.e., staffing, space, materials, leadership, and community linkages) and program quality (i.e., harshness, permissiveness, supportive relations, engagement, and belonging)?
2. What are the demographic program and neighborhood characteristics of the types or profiles?
3. Are types of programs and neighborhood contexts associated with variations in implementation of evidence-based practices in ASPs?

The following methodology describes the sample, measures, and analytical approach to examining these research questions.

## Method

### Sample and Procedures

Data were collected in the context of a cluster randomized control trial of 75 afterschool program sites in a northeastern state including urban, suburban, and rural locales matched by size, socioeconomic status (proportion of children receiving free/reduced lunch), and racial-ethnic distribution and assigned to condition as part of the LEGACY (Leading, Educating, Guiding A Community of Youth) Together Project. We recruited 92% (N = 75) of the program sites, and many providers (N = 12) ran multiple sites (range 2–7). Two programs refused participation due to leadership challenges (i.e., pregnancy and director change), and one did not provide data on critical measures. The current study includes the 72 sites (96% participation rate) with both program- and neighborhood-level information. Table 1 describes the demographic characteristics of the sample and programs which represented rich racial-ethnic, socio-economic, and geographic diversity.

**Table 1:** Demographic description of the programs

Measures	N	Mean	SD	Min	Max
Implementation of evidence-based practices					
ACA—Afterschool Climate Assessment (i.e., Posted behavioral systems and guidelines, praise, reinforcement, incentives, active supervision by adults and interaction with children)	72	.50	.16	.13	.90
Observed program quality variables					
CIS—harshness	72	1.27	.25	1.00	2.42
CIS—permissiveness	72	1.92	.48	1.11	3.26
PPRS—supportive relations with adults	72	3.08	.40	2.07	3.78
PPRS—supportive relations with peers	72	3.23	.31	2.48	3.85
PPRS—level of engagement	72	3.21	.30	2.39	3.85
YPQA—belonging	72	3.40	.43	2.50	4.33
Progam capacity—director report					
Space/facilities	72	2.31	.54	1.27	4.00
Materials	72	2.97	.72	1.60	4.00
Community linkages	72	.01	.55	− .84	1.41
Program leadership	72	.67	.33	0	1.00
Staff capacity and readiness	72	-.03	.83	− 1.61	1.49
Program demographic variables—program archival data					
Average number of children	70	24.61	11.68	11.00	68.00
% free lunch	70	48.46	30.15	1.00	100.00
% White	72	51.88	30.69	0	96.00
% Asian Pacific Islander	72	2.93	3.10	0	17.00
% African-American	72	25.93	25.03	1.00	100.00
% Latino	72	18.13	21.56	0	90.00
Neighborhood context—geographic data					
Urban	20 (28%)				
Suburban	46 (64%)				
Rural	6 (8%)				
Centered diversity	72	.36	.21	.05	.69
Centered risk	72	− .05	.78	− .95	2.06

Observational methods were used to characterize afterschool settings (Oh et al., 2015; Tseng & Seidman, 2007). The training process for the observers was multi-pronged. Initially, observers completed two 8-hour training sessions, which included an overview of constructs and measures, review of videos that illustrated concepts, and scoring practices. In addition to these trainings, observers also participated in “live-practice” in afterschool programs. Observers completed two additional booster trainings before data collection periods. A project staff member was the “Standard Coder” and all observers had to attain 80% agreement through the Gold Standard Video (GSV) process (Stuhlman, Hamre, Downer, & Pianta, 2010). Observers who scored below 80% agreement on any scale were retrained and tested by the Standard Observer before deployment.

Two trained observers (for half of the programs, the other half had one trained observer) collected data on three measures of program quality^1^ (e.g., the Caregiver Interaction Scale—CIS, the Promising Practices Rating Scale—PPRS, and the Youth Program Quality Assessment—YPQA) and one measure of implementation of evidence-based practices (e.g., the Afterschool Climate, Assessment, ACA). Table 2 describes the measures and scales used, their internal consistency (Cronbach’s alpha), and inter-rater reliability (the Interclass Correlation Coefficient, ICC) (Raudenbush & Sadoff, 2008).

**Table 2:** Psychometric and descriptive data for implementation fidelity and afterschool program quality scales

**Implementation fidelity**	**Item N**	**α**	**IRR (ICC)**
**Afterschool Climate Assessment (ACA, index of implementation fidelity)**	10	0.62	0.77
**Observed afterschool program quality scales**			
Caregiver Interaction Scale (CIS)			
Harshness	6	0.75	0.56
Permissiveness	3	0.84	0.58
Promising Practices Rating Scale (PPRS)			
Supportive relations with adults	5	0.88	0.59
Supportive relations with peers	3	0.89	0.50
Level of engagement	3	0.84	0.56
Youth Program Quality Assessment (YPQA)			
Sense of belonging	4	0.55	0.34
**Program capacity measures—director report**			
Space-facilities (e.g., art room, computer lab, kitchen, gym, auditorium, multipurpose room, sports fields and courts)	15	.80	-
Materials (e.g., physical education equipment, books, computers, games, puzzles)	5	.73	-
Community linkages (e.g., frequency and quality of interactions with teachers, parents, and other local resources)	15	.88	-
Leadership—posted mission statement and goals, behavioral management plan in place, rewarding staff who do well	5	.80	-
Staffing capacity and readiness (e.g., eager to learn/develop, responsive to professional development, implementing new curricula and programs, aware and committed to program goals, shared disciplinary practices.)	6	.92	-

In addition to observational methods, survey-based methods were used to assess afterschool program capacity. Afterschool program directors received hard copy surveys from data collection staff to return in the mail. The number of surveys received depended on the number of programs each director led. In the Fall, before the intervention program started, directors’ surveys were collected by research staff.

## Measures

### Quality

Research suggests that multiple measures of quality interactions in educational settings should be employed; as such, we used three observational tools. The 26-item *Caregiver Interaction Scale* (*CIS*, Arnett, 1989) assessed the caregiving styles of afterschool program providers. We used two of the available four subscales in the current study. The *Harshness* subscale examines caustic and insensitive interactions of adults with children. The *Permissiveness* subscale assesses the degree to which staff fail to appropriately provide guidance and redirection when necessary. Observers rated the frequency of these afterschool staff behaviors on a 4-point scale ranging from 1 (never, 0%) to 4 (consistently, > 61%); items were reverse coded with higher scores representing higher quality.

The *Promising Practices Rating Scale* (*PPRS*; Vandell et al., 2004) is an observational measure focused on the quality of specific program activities (e.g., homework assistance, recreation and games, and snack time) used to assess three of the seven original dimensions of program quality (i.e., supportive relationships with adults, supportive relationships with peers, and level of engagement). Each PPRS dimension was rated on a 4-point scale indicating the extent to which each quality was characteristic of the program (where 1 = *highly uncharacteristic*; 2 = *somewhat uncharacteristic*; 3 = *somewhat characteristic*; and 4 = *highly characteristic*). *Supportive relationships with adults* (SRA) assessed the relationship between afterschool staff and students. *Supportive relationships with peers* (SRP) assessed the relationship among afterschool students. *Level of engagement* (LOE) assessed afterschool students’ positive participation in activities.

The *Youth Program Quality Assessment* (YPQA, C. Smith & Hohmann, 2005) can be used to rate individual program offerings (i.e., activities in the program) or the entire program as in this study. The current study used the belonging subscale to examine program belonging, a concept not assessed in the other measures. Belonging was measured by noting the degree to which children were inclusive in their interactions, not excluding other children, exhibiting a common language and gestures as part of their program practice. The YPQA is rated on a 3-point scale using discrete scores of 1, 3, and 5 where 1 indicates that no children have access to this experience; 3, some children have access to this experience; and 5, most children have access to this experience. Higher scores indicate better program quality. This 3-point response scale potentially limits variability in the scale resulting in lower estimates of internal consistency and interrater reliability (Table 2).

### **Capacity**

Capacity was measured with items created for the study and obtained from surveys of the afterschool program directors (N = 32) in the fall prior to program implementation. Some directors completed multiple surveys (n = 13; range = 2–7; mode = 3), because they managed more than one site; 22 directors completed only one survey. The survey was divided into five sections—scales were created from these sections to measure aspects of capacity. The *leadership* scale consisted of five binary items (i.e., yes/no) to assess effective program management. The director’s perceptions of *staff* scale consisted of six items to assess innovation readiness. Directors reported on a 4-point scale ranging from 1 (no staff) to 4 (all staff). The *space* scale consisted of 15 items to examine the availability of important physical rooms and facilities on a 4-point scale—1 (not present), 2 (present but not available), 3 (limited) to 4 (adequate). The *materials* scale examined the availability of important supplies rated on a 4-point scale—1 (not present), 2 (present but not available), 3 (limited) to 4 (adequate). The *community linkages* scale explored the degree of engagement among the afterschool program, parents, schools, and the broader community. Items focused on the frequency of contact (i.e., 6-point scale ranging from 1-never to 6-weekly) between the afterschool program and school teachers and frequency of contact (i.e., 6-point scale ranging from 1-never to 6-weekly) between afterschool program staff and parents. Connections to community organizations ranged from none (1) to five or more (4) that provided support and resources to the afterschool program. Items were averaged for each sub-scale; higher scores indicate greater capacity.

### **Implementation**

The *Afterschool Climate Assessment* (ACA) was created for the research project and assessed the extent to which afterschool providers executed evidence-based practices. These practices included having posted rules and expectations, praise and positive reinforcement, concise directions, and active supervision by adults and interaction with children. Independent observers completed the ACA, a binary checklist of 10 dichotomous yes/no items (α = 0.62); a sum of these 10 items ranged from 0 to 1.00, indicating the proportion of implementation. A higher internal consistency reliability was not expected given that ASPs might implement various aspects of the evidence-based practices assessed.

### **Neighborhood Context**

We explored several indicators of neighborhood context. *Neighborhood urbanicity* was created using the National Center for Educational Statistics metric-centric locale codes (http://nces.ed.gov/ccd/ccdLocaleCode.asp; Phan & Gander, 2008), which is the relative location to a populous area and ranges from city to rural, with eight possible categories. Using the afterschool program site addresses, we located the locale code for that afterschool program site. We condensed the 8 codes into 3 codes (urban, suburban, and rural) for our program sites: 28% were classified as urban (n = 20), 64% were suburban (n = 46), and 8% were classified as rural (n = 6). Afterschool program sites were in 52 census tracts, with an average of 1.38 program sites in each census tract (range = 1–4; mode = 1). Table 1 presents the averages and ranges for the following indicators. *Neighborhood SES* was measured using afterschool program sites addresses, which were geo-coded to obtain data from the 2000 U.S. census on five variables, which have been used in previous studies (Leventhal & Brooks-Gunn, 2000; Witherspoon & Ennett, 2011). *Unemployment* was the percent of unemployed residents in the Labor Force. *Educational attainment* was the percentage of individuals 25 years old or older who had not obtained a high school diploma or GED. *Poverty level* was the percent of residents whose income fell below the poverty level. *Residential mobility* was the percentage of residents who had changed households within the last year. *Female-headed households* was the percentage of female headed households in the tract. These indicators were standardized and averaged to create a *Neighborhood Risk* score with higher scores representing more potential risk.

*Neighborhood diversity* was calculated with Simpson’s Diversity Index (1949; Juvonen et al., 2006), which refers to the diversity of a neighborhood (i.e., census tract) and represents the proportion of residents who self-identified as a member of a racial-ethnic group and the number of racial-ethnic groups represented in that census tract. This index provides an estimate of the probability that any two residents chosen at random will be from different racial-ethnic groups; higher scores indicate greater neighborhood racial-ethnic diversity (range = 0–1).

## Analysis

This study utilized latent profile analysis (LPA) in Mplus 7 to determine if various types of programs were identifiable due to different levels of capacity and program quality. Indicator variables included program director-reported measures of capacity i.e., organizational leadership, staff readiness, resources (e.g., space, materials), community linkages, all at pretest, and program quality (harshness, permissiveness supportive relations with adults/peers, engagement, and belonging), all standardized to facilitate interpretation of the score profiles (Table 1).

Robust maximum likelihood estimator (MLR) was used as it was “designed to be robust against misspecification of the likelihood” for latent variable mixture models (e.g., LPA, LCA) and has been found to perform better than other alternative (variants of ML) methods in standard error estimation under small sample sizes and correct specification of the likelihood (Muthén, 1998–2004, p. 32). Models with increasing number of latent profiles (starting from two) were fit to the data and results were compared on model fit statistics, including information criteria (AIC, Akaike, 1987; BIC, Schwartz, 1978; sample-size adjusted BIC, Sclove, 1987), entropy (Ramaswamy, et al., 1993), Vuong-Lo-Mendell-Rubin likelihood ratio test for k-1 (H0) versus k classes (VLMR-LRT), and Lo-Mendell-Rubin adjusted likelihood ratio test (LMR-adjusted LRT), where k was an integer indicating the current number of profiles being estimated (Lo et al., 2001). Lower information criteria and higher entropy values indicated better fit (e.g., Nylund-Gibson et al., 2014). Entropy values range from 0 to 1, and values close to 1 indicate clear classifications (Muthén, 1998–2004, p. 34). Both VLMR-LRT and LMR-adjusted LRT tested whether the k-profile solution fit better than the k-1-profile solution. A non-significant p-value indicated that the additional latent profile was not necessary because it did not improve model fit significantly (e.g., Nylund-Gibson et al. 2014). In that case, the k-1 profile solution was preferred. Because there is not a single perfect fit statistic that can indicate which model fits best (e.g., Nylund et al., 2007), multiple statistics were considered. Moreover, profile proportions and interpretability of the latent profiles (i.e., distinctiveness of each profile) were also considered when choosing the number of latent profiles (e.g., Muthén & Muthén, 2000).

The association between latent profile membership and each of the neighborhood variables was assessed by either chi-squared test for categorical variables (locale) or by one-way ANOVA for continuous variables (diversity and risk factor). To explore whether the effect of neighborhood context on implementation of evidence-based practices varies by program type (i.e., latent profiles), a series of auxiliary regression models of the distal outcomes program implementation on neighborhood context were estimated using the manual version of the 3-step BCH (Bolck, Croon, & Hagenaar, 2004) method in Mplus to preserve class membership and account for measurement error in the latent class variable (Asparouhov & Muthen, 2014). Four variations of the auxiliary regression model with different constraints across classes were compared: (1) all regression slopes and residual variances were allowed to be different across classes (M1); (2) all regression slopes and residual variances were constrained to be equal across classes (M2); (3) regression slopes were constrained to be equal across classes but residual variances were not (M3); (4) residual variances and the slope for rural (due to small and similar estimates across profiles) were constrained to be equal across classes (M4). The model deemed best fitting based on AIC, BIC, and sample size adjusted BIC was interpreted.

## Results

Our study examines the following research questions: (1) Can we describe different types of programs based on their general capacity (e.g., leadership, communication, staffing, space, community linkages) and program quality (i.e., harshness, permissiveness, engagement, belonging); (2) what are the demographic program and neighborhood characteristics of the types or profiles; and (3) are program types and neighborhood contexts associated with the implementation of evidence-based practices? This section describes the findings of our latent profile analyses.

### Latent Profile Analysis and Capacity

Model fit statistics for 2- to 4-class solutions are given in Table 3. The 4-profile model encountered estimation problems (due to non-positive definite first-order derivative product matrix, standard errors for the estimates of Profile 4 leadership might not be trustworthy) and the solution yielded a very small profile (n = 6). As a result, we did not choose the 4-profile model and did not estimate models with higher number of profiles. All information criteria, AIC, BIC, and sample-size adjusted BIC decreased from 2-profile to 3-profile indicating that the 3-profile solution fit the data better than the 2-profile solution. Entropy was similar between the two models (0.98 vs. 0.96). However, both VLMR-LRT and LMR-adjusted LRT failed to reject the null hypothesis that the 2-profile solution did not fit worse than the 3-profile solution. The profile proportions were quite different for the 2-profile solution (0.21 vs. 0.79). The 3-profile solution appeared to further split the large profile into two yielding profile proportions of 0.22, 0.18, and 0.60. Because both 2- and 3-profile solutions appeared to fit the data quite well (based on VLMR-LRT, LMR-adjusted LRT, and entropy) and there were three distinct patterns of profile scores from the 3-profile solution, we decided to retain three profiles. The three profiles differed significantly on all but one (indicator, namely nonpermissive strategies captured by the CIS quality measure (see the program capacity and quality indicators in Table 4). The first profile, the “Have Nots,” had relatively low initial assessments of director-reported capacity (e.g., leadership, physical space, materials, and staffing) but lower to moderate scores on observed quality (i.e., harshness, criticism, supportiveness, and engagement). The second profile scored higher on *capacity* but demonstrated the lowest observed program quality (e.g., supportiveness, engagement). We termed this, the “Have Some” profile in terms of their scores on capacity. The third profile had high scores on both director-reported capacity scores and observed quality which we referred to as the “Haves.”

**Table 3:** Model fit statistics for 2- to 4-class solutions

Statistics	2-class	3-class	4-class
AIC	2167.38	2092.27	2062.26
BIC	2244.79	2197.10	2194.31
Sample-size adjusted BIC	2137.66	2052.17	2011.57
Entropy	.979	.956	.923
Vuong-Lo-Mendell-Rubin likelihood ratio test for k-1 (H0) vs. k classes	∆2LL = 148.22, df = 12, p = .01	∆2LL = 99.01, df = 12, p = .24	∆2LL = 54.11, df = 12, p = .39
Lo-Mendell-Rubin adjusted likelihood ratio test	Value = 145.39, p = .01	Value = 97.12, p = .25	Value = 53.07, p = .39
Class proportions	.21, .79	.22, .18, .60	.19, .40, .32, .08

**Table 4:** Characteristics of the three latent profiles

Measures	Class 1—Have not (n = 16)		Class 2—Have some (n = 13)		Class 3—Have both (n = 43)	
	Mean	SD	Mean	SD	Mean	SD
Urban	6.3%		30.8%		34.9%	
Suburban	93.8%		53.8%		55.8%	
Rural	0%		15.4%		9.3%	
Index of Neighborhood Diversity (1-D)	.44	.17	.33	.26	.34	.19
Centered Neighborhood Risk Index	− .16	.71	− .07	.77	− .01	.81
Program demographics						
% White children	57.44%	17.56%	59.92%	34.37%	47.37%	33.05%
% African-American children	27.69%	15.58%	22.46%	28.12%	26.33%	27.23%
% Latino children	8.31%	4.98%	14.38%	18.21%	22.91%	24.86%
% Asian Pacific Islander children*	4.50% a	3.22%	1.54% b	1.33%	2.77% ab	3.24%
Average staff years of education*	13.22a	.91	13.99ab	1.26	14.39b	1.34
Average staff age	35.82	8.03	32.60	7.93	35.05	6.71
Average staff social service experience	3.71	2.48	4.91	2.43	4.97	2.70
Average staff child youth experience	4.84	2.60	5.64	2.84	6.21	2.74
% staff male	.10	.19	.17	.18	.21	.26
% staff White	.56	.41	.82	.27	.53	.39
% staff Asian pacific islander or other	.03	.13	.00	.00	.02	.08
% staff Black	.39	.42	.15	.23	.30	.36
% staff Latinx	.02	.06	.03	.08	.14	.28
Number of children enrolled	18.50	6.22	27.42	10.62	26.14	12.88
% free/reduced lunch	37.63	22.53	51.92	29.63	51.60	32.39
Program capacity						
Space**	− .78*a*	.30	.15b	1.15	.24*b*	1.00
Materials***	− 1.56*a*	.38	.56*b*	.70	.41*b*	.57
Community linkages***	− 1.33*a*	.32	.49*b*	.81	.35*b*	.79
Leadership***	− 1.30*a*	.85	.70*b*	.35	.27*b*	.74
Staff**	− .66*a*	.60	.35*b*	.84	.14*b*	1.08
Program quality						
CIS (not harsh)***	− .48*a*	1.11	− .57*a*	1.52	.35*b*	.55
CIS (not permissive)	.01	1.06	− .41	.93	.12	1.00
SRA***	− .28*a*	.81	− 1.39*b*	.84	.53*c*	.60
SRP***	− .29*a*	.88	− 1.18*b*	.61	.46*c*	.81
YPQA—engagement***	− .24*a*	1.03	− 1.11*b*	.91	.43*c*	.72
YPQA—belonging***	− .55*a*	.80	− .90*a*	.60	.48*b*	.88
Implementation						
ACA***	.45*a*	.16	.35*a*	.11	.56*b*	.14

Cell means on the same row that do not share common letters are statistically significant at p < .05 by Tukey’s HSD

^*^p < .05; **p < .01; ***p < .001

### Demographic Program and Neighborhood Characteristics of the Latent Profiles

Table 4 provides detailed descriptions of the three latent profiles’ characteristics, and Table 6 presents statistical test results for differences among profiles. Variables with asterisks indicate statistically significant differences among profiles on them, and cell mean subscripts indicate pairwise comparison results. A substantial proportion of the programs classified as “Have Nots” in terms of capacity were in suburban areas (94%) with 38% of children served receiving free/reduced lunch and served children who were 57% White, 28% Black/African-American, and 8% Latinx, 0.44 in terms of neighborhood diversity (Table 4). The programs characterized as the “Have Some” capacity profile served youth who were 60% White, 22% Black/African-American, 14% Latinx, over half qualified for free/reduced lunch status (52%), and 30–54% were in urban and suburban locales respectively. The programs characterized as the “Haves” profile, demonstrating both capacity and quality, were found across urban (35%), suburban (56%), and to a lesser extent, rural locales, which served a diversity of students in terms of race and ethnicity: 47% White, 26% Black/African-American, and 23% Latinx youth, with a mean of 52% eligible for free/reduced lunch status. The association between profiles and locales was marginally significant (Pearson Chi-square = 8.53, *df* = 4, *p* = 0.07; Fisher-Freeman-Halton exact test value = 8.46, *p* = 0.052). The program profiles did not differ significantly in percentages of different race and ethnicity categories (except % Asian Pacific Islander) or eligibility for free/reduced lunch (see Table 4). The “Have Nots” programs served slightly higher than average % Asian Pacific Islander children than the “Have Some” programs. The “Have” programs’ staff had slightly higher average education than the “Have Nots”.

### Latent Profiles, Neighborhood Context, and Implementation

Among the four auxiliary regression models estimated, model M4 in which residual variance and slope for rural were constrained to be invariant across profiles appeared to fit best based on both AIC and sample size adjusted BIC (see Table 5). Model estimates for M4 are shown in Table 6. Three neighborhood variables were significantly associated with implementation for Profile 1 (the “Have Nots” profile). Specifically, lower diversity, higher risk, and suburban (compared to urban) locale were associated with higher implementation for the “Have Nots” profile. The neighborhood variables did not appear to have statistically significant associations with implementation for Profile 2 (the “Have Some” profile). Diversity was the only neighborhood variable that had a statistically significant association with implementation for Profile 3 (the “Haves” profile), and *higher racial-ethnic* diversity was associated with *higher* implementation. Overall, the “Haves” profile had the highest latent mean on implementation, significantly higher than both the “Have Nots” and “Have Some” profiles.

**Table 5:** Fit statistics for different auxiliary regression models

	AIC	BIC	Sample size adjusted BIC
M1	67.092	110.348	50.486
M2	64.955	87.722	56.215
M3	63.031	90.351	52.543
M4	62.360	98.787	48.376

M1, regression slopes and residual variances were different across classes; M2, regression slopes and residual variances were equal across classes; M3, regression slopes were equal across classes, but residual variances were not; M4, residual variances and the slope for rural were equal across classes

**Table 6:** Auxiliary regression estimates of ACA on neighborhood variables by latent classes

Capacity on	Profile 1 (Have Nots)		Profile 2 (Have Some)		Profile 3 (Haves)	
	Estimate	SE	Estimate	SE	Estimate	SE
Intercept	.339	.221	.217*	.106	.395***	.087
Centered diversity	−.513**	.200	.242	.185	.332*	.133
Centered risk	.214*	.087	-.037	.041	−.021	.038
Rural	.016	.090	.016	.090	.016	.090
Suburban	.401*	.193	.084	.080	.101	.076
Residual variance	.013***	.002	.013***	.002	.013***	.002
Latent means	−1.002***	.297	−1.185***	.327	0^a^	–

Urban is the reference for neighborhood locale

^*^p < .05; **p < .01; ***p < .001

^a^Class 3 is the reference

## Summary and Discussion

 Our study built upon an eco-developmental framework that considers the role of students served, program capacity, and neighborhood context in examining a collection of factors that potentially contribute to the implementation of evidence-based practices found in prior research to result not only in decreased child behavior problems, but also to aspects of positive youth development including a sense of competence, connection, and caring. The ISF framework specifies elements of capacity including internal organizational characteristics such as staffing, communication, and physical resources, along with ways in which they link to the community. Our study adds value by including archival data on the neighborhood geographic and social contexts. With a sample of programs across a northeastern state that varied in racial-ethnic, socioeconomic, and geographic locales, we sought to explore whether types of programs might emerge that could be characterized by capacity and program quality and examine whether their association with implementation of evidence-based practices might vary by neighborhood context.

We found that the “Haves-Nots” were low in terms of director-reported *capacity* (e.g., facilities, space, materials, and staff professional development), but evidenced some lower to moderate level of program quality and relatively higher implementation of evidence-based practices. In this profile, implementation was better in the locales with *more* neighborhood risk (Table 6). The “Have Somes” profile possessed adequate *capacity* but was lowest in quality and implementation. As expected, the “Haves” were programs with high capacity, higher quality, and implementation of evidenced-based practices. The “Haves” spanned urban and suburban areas, serving sizeable proportions of Black and Brown children with a mean of more than half of their children being eligible for free/reduced lunch, an indicator of SES. Implementation was higher among the Haves when sites were more diverse in terms of race-ethnicity (Table 6). Interestingly, the “Have Not” programs with fewer tangible resources in terms of capacity (i.e., space, facilities, staffing) but better in terms of program quality demonstrated greater implementation of evidence-based practices than programs with only capacity (Have Somes), and implementation was better when *more* neighborhood risk was present. Having resources does not appear to be a suitable substitute for the importance of intentional programmatic and social processes that help youth to feel they belong in supportive, engaging community-based afterschool programs.

This study offers some mixed support of the benefit of capacity, instead prioritizing the role of program quality and implementing evidence-based practices. However, as explicated by the ISF, programs that were well-resourced in terms of capacity *and*, with greater quality, excelled relative to the other sites in implementation, speaking to the added value of fostering program capacity. The programs that had capacity and quality implemented well in sites rich in racial-ethnic diversity (Table 6). Returning to the role of neighborhood and community contexts, our eco-developmental approach demonstrated more support for a community asset perspective (McKnight & Kretzmann, 1996) in which the Haves worked to support programs with diverse racial-ethnic children and the Have Nots worked to serve children with higher risk. Interestingly, two of the profiles (Have Somes and Haves) served children similar in terms of racial-ethnic diversity and socioeconomic status, both demonstrating adequate capacity but differing in terms of quality and implementation; the Haves were characterized by capacity, quality, and implementation. These programs with more capacity, quality, and implementation, served children located in urban and suburban communities, diverse in terms of race-ethnicity. The more successful programs were intentional in producing programs high in quality adult and peer relationships and in implementing evidence-based practices, factors that previous research has demonstrated to be critical to children’s socio-emotional and academic outcomes (Smith et al., 2017, 2018; Taylor et al., 2017; Vandell et al., 2018).

These findings should be understood in the context of the strengths and limitations of our sample and measures. Though the participating programs represented a range of children in terms of racial-ethnic and social backgrounds and to some extent geographic locales, the data emanates primarily from a northeastern state and needs replication with a sample with increased range in terms of free-reduced lunch status and neighborhood risk. The average percentage of free/reduced lunch served was 52%, and many programs were characterized as suburban than urban or rural. However, the definition of “suburban” is quite encompassing and varies greatly in terms of the children served in terms of SES and race-ethnicity. The programs in our study represented White, Black/African-American, and to a much lesser extent Latinx and Asian-American youth. Though we worked hard to identify sites including youth diverse in their racial-ethnic backgrounds, other prevention scientists have conducted work focused on Latinx youth in afterschool (Riggs & Greenberg, 2004). More research is needed to examine Asian American, Native, and multi-racial children in afterschool programs. Our observed measures of quality and implementation exhibited moderate to high internal consistency but were lower in interrater reliability. This likely reflects the difficulty, despite bi-monthly booster trainings and reliability checks, to adequately characterize afterschool programs with dozens of staff and children across multiple activities, days of the week and seasons of an academic year. Notwithstanding this limitation, the observed measures of quality still mattered in the analyses. A strength of the study was the use of observational and director-reported data. Capacity, as a concept, though frequently discussed, does not have widely established and utilized measures. We are furthering the research in terms of conceptualizing and assessing capacity. Given our results, building capacity and quality could lead to more optimal results in community youth organizations. Future efforts in understanding capacity might also include more depth in terms of understanding the racial and cultural ecologies of youth programs and ways in which they advance antiracist strategies (Brittian & Williams, 2017; Kendi, 2019; Smith et al., 2017).

In summary, in terms of afterschool, this study contributes to understanding the important role of capacity and implementation of evidence-based practices for quality youth programming. Whereas previous research has suggested and likely is quite true that risk and need can offer barriers to quality, we found that some youth-serving programs can compensate for a lack of resources with a press for higher quality programming; though it is true, that having both is preferable. Interestingly, the staff serving diverse children in terms of race-ethnicity and SES were more experienced and likely possessed the motivation to make a difference for their children. With aspects of “readiness,” i.e., leadership, program structure, this study indicates that geography and diversity need not fatalistically predetermine the success of innovative and intentional program leaders. This research explores how to foster best practices among the young people who need them in terms of their self-regulation, socio-emotional development, and achievement. This is a positive sign for prevention science indicating that efforts to build capacity, and help “the ready and willing” can be fruitful. Further, it is possible that when there is readiness at a higher level of management, it could lead to leadership that builds a cadre of staff who are excited for innovation-specific capacity, to benefit the children they serve. We trust this work begins to offer hope for equitable, innovative practices that help to foster positive youth development across diverse racial-ethnic, socio-economic, and geographic contexts.

## Footnotes

1 See Oh, Osgood, and Smith (2015) for more information about these setting-level (i.e., afterschool program) observations.
